# Application of ^1^H HR-MAS NMR-Based Metabolite Fingerprinting of Marine Microalgae

**DOI:** 10.3390/metabo13020202

**Published:** 2023-01-30

**Authors:** Carolina da Silva Canielles Caprara, Tatiane Ksyvickas Mathias, Maria de Fátima C. Santos, Marcelo G. M. D’Oca, Caroline Da R. M. D’Oca, Fabio Roselet, Paulo Cesar Abreu, Daniela Fernandes Ramos

**Affiliations:** 1 Laboratório de Desenvolvimento de Novos Fármacos (LADEFA), Universidade Federal do Rio Grande (FURG), Rio Grande 96200-400, RS, Brazil; 2 NMR Laboratory, NMR Center, Departamento de Química, Universidade Federal do Paraná, Curitiba 81530-900, PR, Brazil; 3 Laboratório de Produção de Microalgas (LPM), Instituto de Oceanografia, Universidade Federal do Rio Grande (FURG), Rio Grande 96210-030, RS, Brazil; 4 Núcleo de Desenvolvimento de Novos Fármacos—NUDEFA, Rua General Osório, s/n°, Campus Saúde, 2° andar, Rio Grande 96200-400, RS, Brazil

**Keywords:** microalgae, coastal products, HR-MAS NMR, metabolomics, biotechnology, bioactivity

## Abstract

Natural products from the marine environment as well as microalgae, have been known for the complexity of the metabolites they produce due to their adaptability to different environmental conditions, which has been an inexhaustible source of several bioactive properties, such as antioxidant, anti-tumor, and antimicrobial. This study aims to characterize the main metabolites of three species of microalgae (*Nannochloropsis oceanica*, *Chaetoceros muelleri,* and *Conticribra weissflogii*), which have important applications in the biofuel and nutrition industries, by ^1^H High-resolution magic angle spinning nuclear magnetic resonance (^1^H HR-MAS NMR), a method which is non-destructive, is highly reproducible, and requires minimal sample preparation. Even though the three species were found in the same ecosystem and a superior production of lipid compounds was observed, important differences were identified in relation to the production of specialized metabolites. These distinct properties favor the use of these compounds as leaders in the development of new bioactive compounds, especially against environmental, human, and animal pathogens (One Health), and demonstrate their potential in the development of alternatives for aquaculture.

## 1. Introduction

Oceans cover 70% of the planet’s surface and contain about 97% of its water [1]. Marine and coastal environments cover a diverse spectrum of habitats, from shallow coral reefs to deep hydrothermal vents, and are home to an enormous diversity of species, from micro- to macro-organisms. This species diversity is translated into unique metabolic abilities to ensure survival in harsh marine conditions [2]. Compared to terrestrial environments, marine and coastal environments favor the production of natural products, as approximately 28,500 marine natural products were identified by the end of 2016, many with a high degree of structural novelty [3]. 

Microalgae are unicellular organisms that convert carbon dioxide, light, water, and macro- and micronutrients into biomass, mainly carbohydrates, lipids, proteins, and pigments. Recent research estimated a diversity of 72,500 species of algae, of which 39% are yet to be described [4]. Microalgae live in close relation with bacteria and predators; therefore, microalgae have developed several chemical defense mechanisms [3]. They are adapted to a wide range of environmental conditions, which influence the production of metabolites [5], which are a potential source of marine natural products [6]. 

Potential advantages of using microalgae as a raw material for various purposes in biotechnology include its ability to synthesize and accumulate large amounts of neutral lipids. These lipids are composed of a large percentage of polyunsaturated fatty acids that are important for the diet of other aquatic organisms and for human health [7]. Furthermore, fatty acids have great commercial potential when incorporated into food, animal feed, cosmetics, and pharmaceutical products [8]. Products from marine organisms have shown many interesting activities, such as antimicrobial, cytotoxic, anticancer, antidiabetic, antifungal, anticoagulant, anti-inflammatory, and other pharmacological activities [9,10,11].

Interest in the biotechnological potential of microalgae has increased due to their high production rates and their production of several valuable substances [12]. The cultivation of microalgae has gained great momentum among researchers in recent decades due to their ability to produce valuable metabolites, remarkable photosynthetic efficiency, and versatile nature [13]. In Brazil, the government has promoted a national strategy to encourage and strengthen in vitro studies that validate the use of metabolites produced by microalgae and other marine organisms in different aquaculture systems. The biomass produced will be used as a raw material for the production of many biomolecules used in the food and pharmaceutical industries [14]. In this scenario, we highlight *Nannochloropsis oceanica*, a unicellular microalga that belongs to the class Eustigmatophyceae that has high biotechnological potential due to its massive lipid production [15,16,17,18] and the diatoms of the genus Chaetoceros and Conticribra, which are among the most cultivated diatoms for larviculture [19]. Diatoms belong to a large group of microalgae and are among the most abundant classes in marine environments [20]. The microalgae *Chaetoceros muelleri* is used due to its fatty acid profile, and the diatom *Conticribra (Thalassiosira) weissflogii* is an euryhaline species [21] and has high carbon fixation rates, as well as excellent photosynthetic efficiency. Both have been used as a model in physiological studies of photosynthesis [22,23]. The lipid content of *C. weissflogii* was analyzed, mainly as a source of fatty acids for copepods [24]. 

The methodologies commonly used to study the chemical profile of microalgae are laborious and require extraction with organic solvents. An alternative that has been used to directly investigate the chemical composition of microalgae preserving the integrity of the compounds is the High-resolution magic-angle spinning nuclear magnetic resonance spectroscopy (HR-MAS NMR) [25,26,27]. The HR-MAS NMR has been used for investigations based on the fingerprints or metabolomic profiles of biofluids [27], herbal medicines [28,29], foods [30], and microalgae [31]. This technique allows the analysis of semi-solid samples that are characterized by inhomogeneity and restricted molecular motion with spectral resolution similar to the solution-state NMR [25,26]. This happens due to the molecular mobility in the swollen samples, which is associated with fast spinning around of the so-called “magic angle” (θ = 54.74°). As a result, line broadening is drastically reduced due to dipole coupling and chemical shift anisotropy (CSA) [26]. The main advantage of the HR-MAS NMR is that it allows the identification of the metabolic profile under conditions similar to the natural chemical environment of the compounds with the same spectral resolution of NMR solution. 

Several studies have demonstrated the abundance of primary metabolites produced by microalgae; these metabolites include lipids, fatty acids, amino acids, and some sugars. However, there are few studies that investigate the complex characterization of marine microalgae, especially through more robust tools, and that enable fast, easy, non-destructive, and unbiased screening such as ^1^H NMR-based metabolomics [32,33,34,35]

Carotenoids, polyphenols, and polyunsaturated fatty acids (PUFAs) have gained prominence among the bioactive compounds produced by microalgae [36,37,38], which have been widely proposed for biotechnological use in the area of food, fertilizers, bioenergy, cosmetics, pharmaceuticals, and industries in general [39]. 

In this way, the aim of the present study was to evaluate the spectral profiles of microalgae *N. oceanica, C. muelleri,* and *C. weissflogii* microalgae in polar and non-polar solvents by ^1^H HR-MAS NMR to provide a more comprehensive investigation of these microalgae that can contribute to the targeting of applications in several areas.

## 2. Materials and Methods

### 2.1. Microalgae Cultivation

The microalgae used in this study, namely *Nannochloropsis oceanica* Suda and Miyashita 2002 (Eustigmatophyceae), *Chaetoceros muelleri* Lemmermann 1898 (Mediophyceae), and *Conticribra weissflogii* (Grunow) Stachura-Suchoples and D.M. Williams 2009 (Mediophyceae), were obtained from the collection of the Laboratory of Microalgae Production from the Institute of Oceanography, Federal University of Rio Grande (FURG/Brazil). The microalgae were cultivated in natural seawater (salinity 28), which was measured using a hand-held salinity refractometer (ATAGO, Bellevue, WA, USA). The seawater was first filtered by a sand filter. Next, it was further filtered through a 1.0 µm polypropylene filter cartridge, disinfected with sodium hypochlorite 12% (NaClO, 0.15 mL L^−1^) for 24 h, and then neutralized with ascorbic acid (C_6_H_8_O_6_, 30 mg L^−1^). Batch culture technique was employed, using Guillard’s medium [32], composed of sodium nitrate (NaNO_3_, 75 mg L^−1^), sodium phosphate (NaH_2_PO_4_·H_2_O, 5 mg L^−1^), EDTA (C_10_H_14_N_2_O_8_Na_2_·2H_2_O, 4.36 mg L^−1^), ferric chloride (FeCl_3_·6H_2_O, 3.15 mg L^−1^), cupric sulfate (CuSO_4_·5H_2_O, 0.01 mg L^−1^), zinc sulfate (ZnSO_4_·7H_2_O, 0.02 mg L^−1^), cobalt chloride (CoCl_2_·6H_2_O, 0.01 mg L^−1^), manganese chloride (MnCl_2_·4H_2_O, 0.18 mg L^−1^), sodium molybdate (Na_2_MoO_4_·2H_2_O, 0.006 mg L^−1^), thiamine (C_12_H_17_ClN_4_OS·HCl, 0.1 mg L^−1^), cyanocobalamin (C_63_H_88_CoN_14_O_14_P, 0.0005 mg L^−1^), and biotin (C_10_H_16_N_2_O_3_S, 0.0005 mg L^−1^). Diatoms were supplemented with sodium silicate (Na_2_SiO_3_·9H_2_O, 30 mg L^−1^). All chemicals were analytical grade (Dinâmica, Indaiatuba, São Paulo, Brazil). The microalgae were cultured in a 330 L bubble column acrylic photobioreactor (0.5 m diameter, 1.5 m height), and kept indoors under controlled conditions (23 °C, 150 µmol m^−2^ s^−1^, and a light period of 16h L: 8h D). The culture growth was monitored daily by counting the cell abundance using an improved Neubauer hemocytometer (Marienfeld, Lauda-Königshofen, Baden-Württemberg, Germany), and cultivation was maintained for 12 days until the stationary phase was reached.

### 2.2. Biomass Harvesting

The microalgae were harvested by flocculation using Flopam FO 4800 SH, an organic synthetic polyacrylamide-based polymer [34]. The flocculant was prepared by adding 3.3 g of polymer to 3.3 L of deionized water and by mixing at 300 rpm until dissolution (ca. 1 h). Then, the flocculant solution was poured into the photobioreactor. Concurrently, the microalgae suspension was mixed intensively (100 rpm) for 5 min to allow uniform flocculant dispersal, followed by gentler mixing (20 rpm) for 15 min to allow floc formation. The flocculated biomass settled within 10 min and was collected after the supernatant was siphoned. The biomass was further dewatered by centrifugation (2527× *g*) at 4 °C for 5 min, and then dried at 60 °C for 24 h.

### 2.3. Sample Preparation for ^1^H HR-MAS NMR

An amount of 10 mg of dried marine microalgae was inserted into the zirconia rotor (50 µL), then 40 µL of deuterated solvent was added, homogenized, and packed. The solvents used were chloroform (CDCl_3_) and methanol (CD_3_OD), both at 0.05% of TMS, and water (D_2_O) at 0.01% of TMSP.

### 2.4. Acquisition of ^1^H HR-MAS NMR Spectra

The ^1^H HR-MAS NMR spectra were acquired on a Bruker AVANCE 400 NMR spectrometer (Bruker, Karlsruhe, Germany), which was operated at 9.4 T, with observation of the ^1^H core at 400.13 MHz, and which was equipped with a quadrinuclear probe (^1^H/^13^C/^15^N/^2^H) at a high resolution with 4mm magic angle (θ = 54.74°) rotation and field gradient in the magic angle direction. The ^1^H HR-MAS NMR spectra were acquired with the *zgpr* pulse sequence, a recycle delay (D1) of 1.0 s, and 64 transients and 64k points (TD) distributed in a spectral window of 8012.820 Hz, resulting in time acquisition (AQ) of 4.01 s and a temperature of 298 K. During acquisition, the samples were rotated at a speed of 5 KHz around the magic angle.

The 1D and 2D NMR spectra were obtained at 298 K in a AVANCE III NMR spectrometer (Bruker, Karlsruhe, Germany) which operated at 14.7 T (^1^H at 600.13 MHz and ^13^C at 150.92 MHz) and was equipped with a TCI CryoProbe Prodigy 5 inverse detection probe with a z-gradient. Tuning (matching and tuning) for ^1^H and ^13^C and adjustment of the homogeneity of the magnetic field spatially (shimming) were performed for each analysis.

### 2.5. Sample Preparation and Acquisition of 1D and 2D NMR Spectra

To support NMR chemical shift assignments, experiments of 1D and 2D NMR in solution with *N. oceanica, C. muelleri,* and *C. weissflogii* were also performed. For this purpose, 100.0 ± 1.0 mg of each sample was submitted to extraction directly in 1 mL of CDCl_3_, D_2_O, and CD_3_OD sonicated and subsequently centrifuged at 12,000 rpm for 10 min. After this procedure, 600 µL of the extracts were added to 5 mm tubes for further analyses. Direct and long-range ^1^H-^13^C NMR correlations were acquired on a Bruker Avance III 600 NMR spectrometer operating at 14.1 T and observing ^1^H and ^13^C at 600.13 and 150.92 MHz, respectively. The spectrometer was equipped with a 5 mm inverse detection four-channel probe (^1^H, ^13^C, ^15^N and ^31^P) with z-gradient. One-bond and long-range ^1^H-^13^C correlations from HSQC and HMBC NMR experiments were optimized for average coupling constants ^1^J_(H,C)_ and ^LR^J_(H,C)_ of 140 and 8 Hz, respectively.

## 3. Results

**Figure 1:**
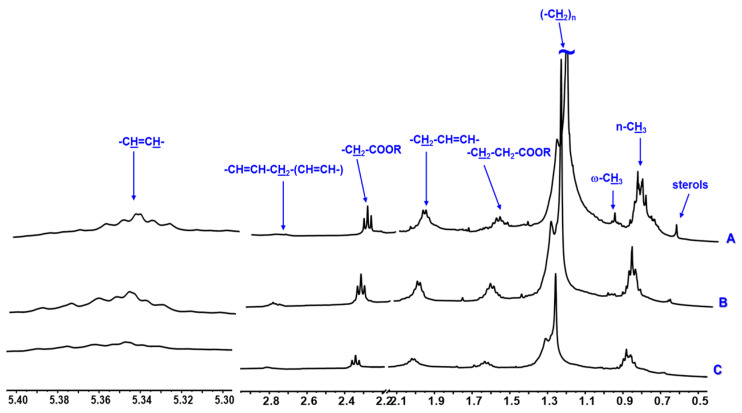
^1^H HR-MAS NMR spectra (400 MHz, CDCl_3_) of the microalgae *N. oceanica* (A), *C. muelleri* (B), and *C. weissflogii* (C). In blue, types of hydrogens of polyunsaturated fatty acids are indicated.

**Figure 2:**
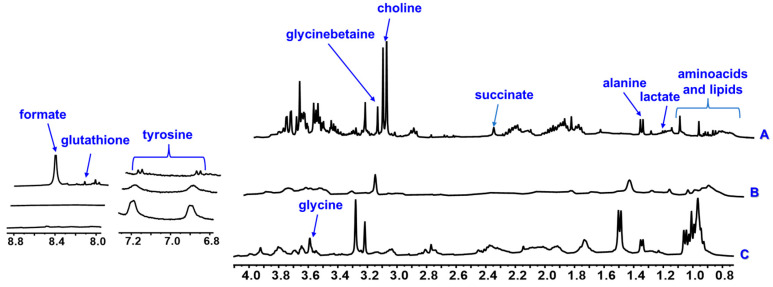
^1^H HR-MAS NMR spectra (400 MHz, D_2_O) of the microalgae *N. oceanica* (A), *C. muelleri* (B), and *C. weissflogii* (C). In blue, the metabolites identified are indicated.

**Figure 3:**
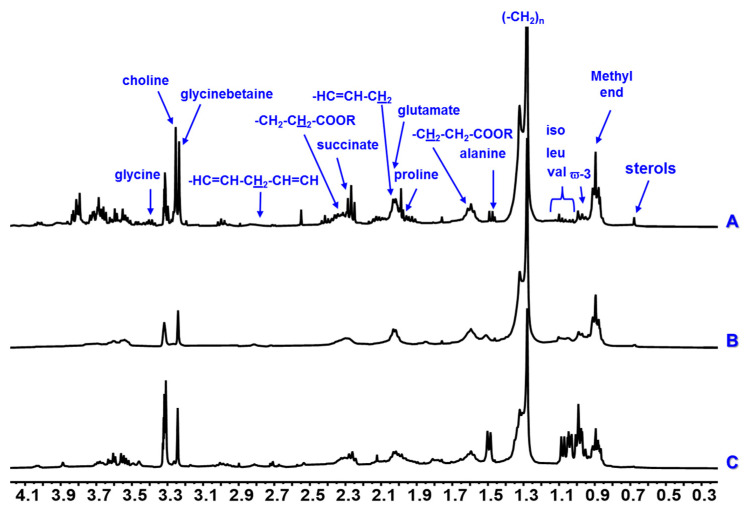
^1^H HR-MAS NMR spectra (400 MHz, MeOD) of the microalgae *N. oceanica* (A), *C. muelleri* (B) and *C. weissflogii* (C). In blue, the metabolites or types of hydrogens identified are indicated.

Initially, the metabolite fingerprinting of the marine microalgae *N. oceanica, C. muelleri,* and *C. weissflogii* were explored by ^1^H HR-MAS NMR. Due to restricted and low molecular motion conditions of semi-solid samples such as microalgae, several anisotropic trends such dipolar interactions, magnetic susceptibility, and chemical shift anisotropy affect T2 relaxation, producing a line-broadening of signals, low signal-to-noise ratio, and low resolution in the NMR spectra. The high spinning rates of the samples, which occur at a magical angle (θ = 54.74°), minimize the line-broadening effects coming from dipolar interactions. Additionally, the HR-MAS technique is applied in swollen samples, employing suitable NMR solvents, which provide sufficient molecular motions in order to improve spectral resolution. In this sense, to have a more comprehensive view of the mobility of microalgal compounds, ^1^H HR-MAS NMR spectra were recorded directly from samples by employing 40 µL of chloroform, methanol, or water as solvents (Figure 1, Figure 2 and Figure 3) without sample pretreatment steps and then preventing changes in the chemical compositions during extraction and isolation procedures.

According to the ^1^H HR-MAS NMR spectra of marine microalgae *C. muelleri*, *C. weissflogii*, and *N. oceanica* analyzed in deuterated chloroform (Figure 1), it was possible to observe that the three species of microalgae presented a similar chemical profile because they are constituted of mono- or polyunsaturated fatty acids. From the ampliation of the region between δ_H_ 5.40–0.50, characteristic signals of vinylic hydrogens were observed at δ_H_ δ_H_ 5.40–5.30 (*m*), bis-allylic at δ_H_ 2.81 (*m*), α–C=O in δ_H_ 2.34 *t* (*J* = 7.5 Hz), allylic at δ_H_ 2.06 (*m*), β–C=O in δ_H_ 1.62 (*m*), methylene at δ_H_ 1.35–1.25(*m*), and methyls at δ_H_ 0.87 (*m*). Only for the microalgae *N. oceanica,* a triplet at δ_H_ 0.98 (*J* = 7.0 Hz) relative to methyl hydrogens of type ω-3 was observed.

The spectral profile acquired directly from dried marine microalgae samples using deuterated water (or D_2_O) as the solvent seemed to be more complex, with several signals overlapped; thus, it was difficult to clearly identify the signals from the chemical compounds (Figure 2). The entire signal assignments of *C. muelleri*, *C. weissflogii,* and *N. oceanica* were confirmed based on 2D NMR experiments, which were performed in solution state (Figures S1–S13), as well as previously reported data [35]. From these spectra, it was possible to identify eight chemical compounds, including amino acids and organic acids. Regarding amino acids, a doublet at δ_H_ 1.47 (J = 7.2 Hz) was observed for the methyl hydrogen of alanine for the microalgae *N. oceanica* and *C. weissflogii.* On the other hand, for *C. muelleri,* a broad simplet at δ_H_ 1.49 was observed due to the lower spectral resolution for this microalga. The three microalgae presented a simplet at δ_H_ 3.59 relative to the methine hydrogen of glycine. 

In the three microalgae, typical relative signals of hydrogens from the para-substituted aromatic ring of tyrosine were observed. However, for *N. oceanica,* these signals presented with higher spectral resolution because a two doublet was observed at δ_H_ 6.89 (*J* = 8.1 Hz) and 7.19 (*J* = 8.1 Hz). Furthermore, it was also observed that only *C. weissflogii* presented a simplet at δ_H_ 8.23 relative to the N-H hydrogen of the tripeptide cysteine amino acid, glutathione. Furthermore, organic acids have also been identified. A doublet at δ_H_ 1.33 (*J* = 6.7 Hz) was observed for the methyl lactate hydrogens for the three microalgae, and a simplet was observed at δ_H_ 2.40 for the succinate methylene hydrogens only for Figure 2. A simplet at δ_H_ 3.20 relative to the methyl hydrogens of the choline was observed for the three microalgae in question. A signal at δ_H_ 8.47 (*s*) was also observed relative to the methynic hydrogen of the formate only for *N. oceanica*. The attributions of these metabolites in comparison with the literature [35] are presented in Table 1.

From the spectra acquired employing methanol, it was possible to identify a greater number of compounds from different classes, indicating that methanol promoted greater molecular mobility. In addition, it was observed that with this solvent, the spectral profiles presented a better spectral resolution. Thus, signals were observed in the regions of aliphatic hydrogens, amino acids, sugars, and aromatics (Figure 3). The attributions of these metabolites by 1D and 2D NMR and in comparison with the literature [35,40,41] are presented in Table 1.

## 4. Discussion

In this study, the spectra revealed the presence of different classes of metabolites including amino acids and mono- and polyunsaturated fatty acids and organic acids. In addition, 13 amino acids were identified in at least one of the samples evaluated using water or methanol as solvents in spectral regions compatible with the description in the literature, ranging 0.50–8.50 ppm [35,42]. The choice of solvent used to swell the samples showed great importance for identifying the compounds present in the microalgae matrix. Spectra recorded with the addition of methanol and water, for example, were able to increase molecular mobility and, thus, result in more defined and resolved signals that identified more compounds than chloroform, with amino acids and organic acids emerging in the spectra. However, monounsaturated fatty acids and PUFAs were identified more frequently with chloroform.

Some compounds seem to be characteristic markers according to the species of microalgae analyzed, with *N. oceanica* showing the greatest diversity of compounds, followed by *C. weissflogii* and *C. muelleri*. We emphasize that, unlike other species of the genus Nannochloropsis, *N. oceanica* has been described as having mostly lipids and proteins, which represent approximately half of the dry weight of the biomass, according to Zanella and Vianello (2020). This genus of microalgae has high photosynthetic efficiency and can convert carbon dioxide to storage lipids mainly in the form of triacylglycerols and to the ϖ-3 long-chain polyunsaturated fatty acid eicosapentaenoic acid (EPA) [17]. Interestingly, *N. oceanica* was the only coastal species evaluated that presented, at 8.47 s, a characteristic peak of formate. Inhibition of the folate biosynthetic pathway, which may have formate as a product, has been proposed as a promising target for the development of several antimicrobial compounds with cytotoxic activity against tumor cells, for example [43,44,45]. In addition, formate can act as a by-product of anaerobic fermentation of some bacterial species and interfere, as a secondary metabolite, in different pathophysiological conditions.

With regard to fatty acids, which are already widely recognized as important metabolites present and biotechnologically important in microalgae of the genus Nannochloropsis, and EPA and PUFAS [37,42], which are the major fatty acids in *N. oceanica*, only this microalgae presented omega-3 fatty acids (ϖ-3), and this metabolite is referenced by many authors as beneficial to human and animal health, as well as in the pharmaceutical, food, environmental, and cosmetic industries [17,42].

On the other hand, recent studies have proposed that α-linolenic acid (ALA), EPA, and docosahexaenoic acid (DHA) are major bioactive compounds in different species of the genus Chaetoceros [46]. 

Surprisingly, among the evaluated polyunsaturated fatty acids in the metabolomics analyses for *C. muelleri*, only methylic ϖ-3 (0.98 *t*) was not identified, contrary to the findings of Ramos et al (2022), which identified potential antibacterial activity in extracts of different polarities which contained this metabolite through ^1^H Nuclear Magnetic Resonance (NMR) analysis. 

To the best of our knowledge, this is the first study that characterizes metabolites through assays with ^1^H HR-MAS NMR of *C. weissflogii*, which is a model species for environmental studies and in the development of energy and feed supplements for animals [47,48]. Since before its reclassification (previously known as *Thalassiosira weissflogii*) [48,49], this species has demonstrated important cytotoxic and immunomodulatory bioactivity related to the presence of polysaccharides, as well as the important production of metabolites that are toxic against different microorganisms; for example, it interferes with *quorum sensing* and the adhesion and formation of bacterial biofilms [47,50,51].

Therefore, metabolomics studies of species belonging to coastal ecosystems—such as microalgae, which suffer intensely from abiotic factors such as constant changes due to seasonal heat input and thermal stratification, as well as ecosystem variations—are increasingly necessary. Such studies are necessary in order to understand specific responses of organisms to abiotic stressors and the organism–environment interactions and between the communities that constitute this niche [52].

In this sense and in corroboration with the objectives of the Brazilian Biotecmar [14] strategy, innovative, technological strategies are necessary to favor marine biotechnology as a whole. These strategies would aid in the understanding of the impacts of each individual that comprises this ecosystem on the biosphere, the monitoring of the impacts of environmental disturbances, and the use of bioactive metabolites for the development of therapeutic products in favor of sustainable development and the maintenance of One Health.

## 5. Conclusions

The fingerprinting approach of coastal microalgae by HR-MAS NMR proved to be an interesting tool to identify metabolites with minimal sample preparation that may contribute to future investigations of unpublished compounds or little explored compounds, which can contribute to the development of biotechnological products. Marine microalgae, such as the diatom *Conticribra weissflogii*, the major chemical composition of which has been described for the first time in this study, have highlighted as a fundamental element in the food chains of aquatic ecosystems, in addition to its use in the production of food and new drugs. In addition, our study allowed us to identify different metabolite profiles among the three evaluated species, evidencing the possibility of these marine microalgae to act as raw materials for bioactive compounds applied to *One Health*, the food industry, animal production, and cosmetics. Lastly, the results demonstrate that methanol was the best solvent; however, it must be noted that the choice in the solvent depends on the type of compound ones wants to extract.

## Figures and Tables

**Table 1:** Assignment of main resonances of marine microalgae by ^1^H HR-MAS NMR (400 MHz).

Class	Compound	δ_H_ mult. (*J* in Hz)	CDCl_3_			D_2_O			CD_3_OD		
			N. O	C. M	C. W	N. O	C. M	C. W	N. O	C. M	C. W
Amino Acids	Isoleucine	0.95 *t* (7.0), 1.02 *d* (7.0)	-	-	-	x	x	x	x	x	x
	Leucine	0.99 *m*	-	-	-	x	x	x	x	x	x
	Valine	1.02 *d* (7.0), 1.07 *d* (7.0)	-	-	-	x	x	x	x	x	x
	Alanine	1.48 *d* (7.0)	-	-	-	x	x	x	x	x	x
	Proline	1.97–2.07 *m*	-	-	-	-	-	-	x	x	x
	Glutamate	2.08 *m*, 2.14 *m*, 2.35 *m*	-	-	-	-	-	-	x	x	x
	Choline	3.20 *s*	-	-	-	x	x	x	x	x	x
	Glycinebetaine	3.26 *s*	-	-	-	x	x	x	x	x	x
	Glycine	3.59 *s*	-	-	-	x	x	x	x	x	x
	Phenylalanine	7.24 *m*, 7.30 *m*, 7.34 *m*	-	-	-	x	x	x	x	-	x
	Histidine	7.24 *m*, 8.13 *s*	-	-	-	-	-	-	x	-	x
	Tyrosine	6.89 *d* (8.1), 7.19 *d* (8.1)	-	-	-	x	x	x	x	x	x
	Glutatione	8.23 *s*	-	-	-	x	x	x	-	-	-
Organic acids	Lactate	1.33 *d* (6.7)	-	-	-	x	x	x	-	-	-
	Succinate	2.30 *s*	-	-	-	x	-	x	x	x	x
	Formate	8.47 *s*	-	-	-	x	-	-	x	-	-
	Sterols	0.73 *m*	x	x	x	-	-	-	x	x	x
Monounsaturated Fatty acids	Methylic	0.89 *m*	x	x	x	-	-	-	x	x	x
	Methylenic	1.35–1.25 *m*	x	x	x	-	-	-	x	x	x
	–CH_2_–CH_2_–COOR	1.59 *m*	x	x	x	-	-	-	x	x	x
	–CH_2_–CH_2_–COOR	2.34 *m*	x	x	x	-	-	-	x	x	x
PUFAs	Methylic ϖ-3	0.98 *t* (7.0)	x	-	-	-	-	-	x	-	-
	–CH_2_–CH_2_–COOR	1.62 *m*	x	x	x	-	-	-	x	x	x
	Allylic	2.06 *m*	x	x	x	-	-	-	x	x	x
	–CH_2_–CH_2_–COOR	2.34 *m*	x	x	x	-	-	-	x	x	x
	*bis*-allylic	2.81 *m*	x	x	x	-	-	-	x	x	x
	Vinylic	5.40–5.30 *m*	x	x	x	-	-	-	x	x	x

N. O—*N. oceanica*; C. M—*C. muelleri*; C. W—*C. weissflogii*. (δ) Chemical shift in ppm. x—Indicates the presence of the metabolite.

## Data Availability

The data presented in this study are available in Figure 1, Figure 2 and Figure 3, Table 1, and Figures S1–S4 in the Supplementary Material.

## Supplementary Materials

The following supporting information can be downloaded at: https://www.mdpi.com/article/10.3390/metabo13020202/s1, Figure S1. ^1^H-^13^C one-bond correlation map from multiplicity edited HSQC NMR experiment acquired from *Conticribra weissflogii* (^1^H and ^13^C, 600 and 150 MHz, D_2_O)—regions A, B, and C; Figure S2. Ampliation from ^1^H-^13^C one-bond correlation map from multiplicity edited HSQC NMR experiment acquired from *Conticribra weissflogii* (^1^H and ^13^C, 600 and 150 MHz, D_2_O)—region *A;* Figure S3. Ampliation from ^1^H-^13^C one-bond correlation map from multiplicity edited HSQC NMR experiment acquired from *Conticribra weissflogii* (^1^H and ^13^C, 600 and 150 MHz, D_2_O)—region *B;* Figure S4. Ampliation from ^1^H-^13^C one-bond correlation map from multiplicity edited HSQC NMR experiment acquired from *Conticribra weissflogii* (^1^H and ^13^C, 600 and 150 MHz, D_2_O)—region *C*; Figure S5. ^1^H-^13^C one-bond correlation map from multiplicity edited HSQC NMR experiment acquired from *Chaetoceros muelleri* (^1^H and ^13^C, 600.13 and 150.92 MHz, CD_3_OD)—regions A, B, and C; Figure S6. Ampliation from ^1^H-^13^C one-bond correlation map from multiplicity edited HSQC NMR experiment acquired from *Chaetoceros muelleri* (^1^H and ^13^C, 600.13 and 150.92 MHz, CD3OD)—region *A;* Figure S7. Ampliation from ^1^H-^13^C one-bond correlation map from multiplicity edited HSQC NMR experiment acquired from *Chaetoceros muelleri* (^1^H and ^13^C, 600.13 and 150.92 MHz, CD_3_OD)—region *B*; Figure S8. Ampliation from ^1^H-^13^C one-bond correlation map from multiplicity edited HSQC NMR experiment acquired from *Chaetoceros muelleri* (^1^H and ^13^C, 600.13 and 150.92 MHz, CD_3_OD)—region *C;* Figure S9. ^1^H-^13^C long-distance correlation map HMBC NMR experiment acquired from *Chaetoceros muelleri* (^1^H and ^13^C, 600.13 and 150.92 MHz, CD_3_OD); Figure S10. ^1^H-^13^C one-bond correlation map from multiplicity edited HSQC NMR experiment acquired from *Nannochloropsis oceanica* (^1^H and ^13^C, 600.13 and 150.92 MHz, CD_3_OD)—regions A, B, and C; Figure S11. Ampliation from ^1^H-^13^C one-bond correlation map from multiplicity edited HSQC NMR experiment acquired from *Nannochloropsis oceanica* (^1^H and ^13^C, 600.13 and 150.92 MHz, CD_3_OD)—region *A*; Figure S12. Ampliation from ^1^H-^13^C one-bond correlation map from multiplicity edited HSQC NMR experiment acquired from *Nannochloropsis oceanica* (^1^H and ^13^C, 600.13 and 150.92 MHz, CD_3_OD)—region *B*; Figure S13. Ampliation from ^1^H-^13^C one-bond correlation map from multiplicity edited HSQC NMR experiment acquired from *Nannochloropsis oceanica* (^1^H and ^13^C, 600.13 and 150.92 MHz, CD_3_OD)—region *C*.
