# Supplementary material for: Application of 1H HR-MAS NMR-Based Metabolite Fingerprinting of Marine Microalgae
Source: Metabolites. 2023 Jan 30;13(2):202. doi: 10.3390/metabo13020202 (PMC9965007; doi:10.3390/metabo13020202)
Supplement: Supplementary file 1 [file metabolites-13-00202-s001.zip › metabolites-2150250-supplementary.pdf]

Supplementary Files

Application of ^{1}H HR-MAS NMR-based metabolite fingerprinting of marine microalgae

Carolina da Silva Canielles Caprara [1], Tatiane Ksyvickas Mathias [2], Maria de Fátima C. Santos [2], Marcelo G. M. D'Oca [2], Caroline Da R. M. D'Oca [2], Fabio Roselet [3], Paulo Cesar Abreu [3†] and Daniela Fernandes Ramos [1,*]

†This work is dedicated to the memory of our dear colleague and mentor, Professor Paulo Cesar Abreu, who passed away on July 19, 2022.

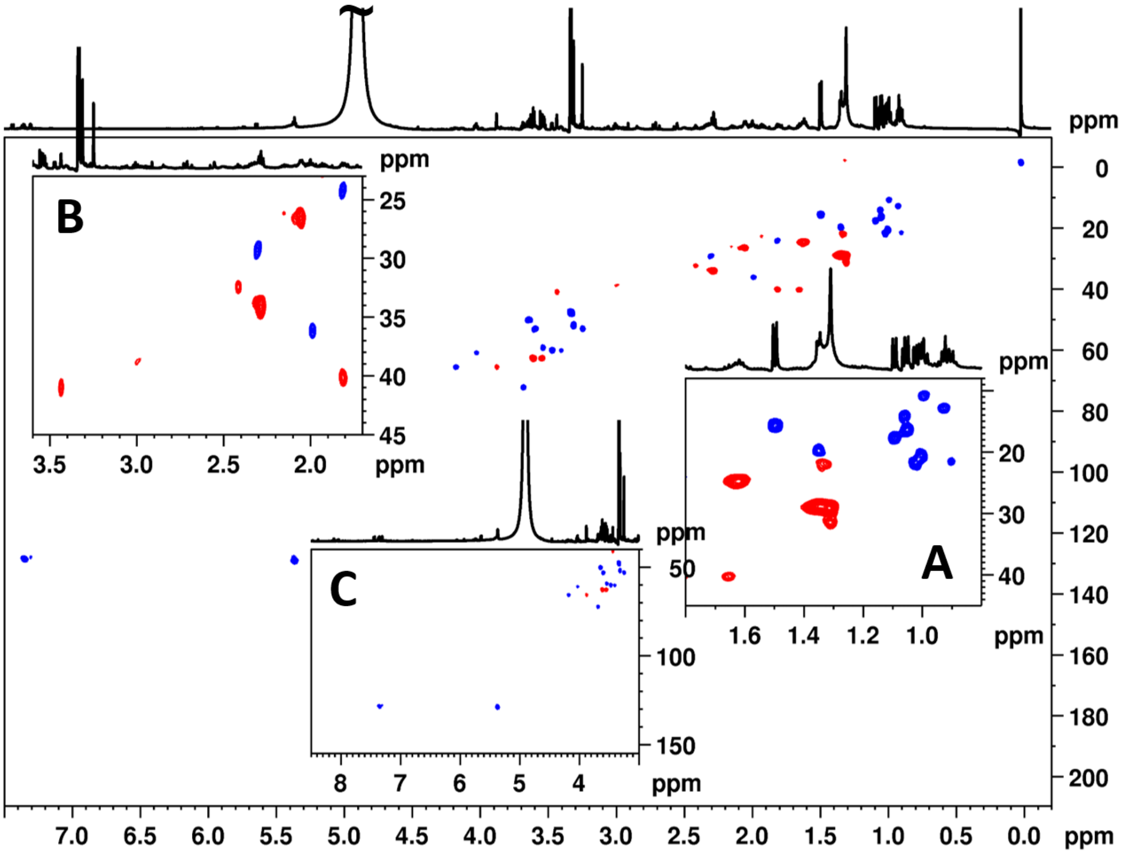

Figure S1. ^{1}H-^{13}C one-bond correlation map from multiplicity edited HSQC NMR experiment acquired from *Conticribra weissflogii* (^{1}H and ^{13}C, 600 and 150 MHz, D_2O) – regions A, B, and C.

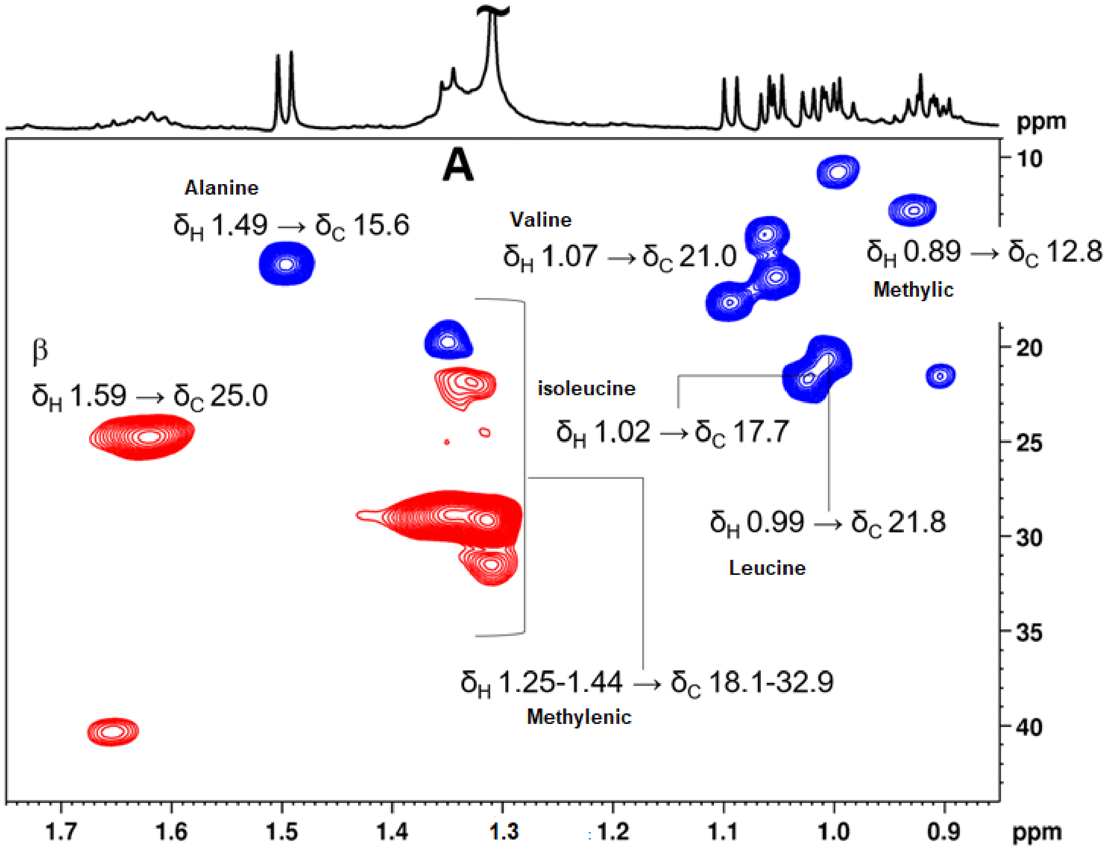

Figure S2. Ampliation from ^{1}H-^{13}C one-bond correlation map from multiplicity edited HSQC NMR experiment acquired from *Conticribra weissflogii* (^{1}H and ^{13}C, 600 and 150 MHz, D_2O) – region *A.*

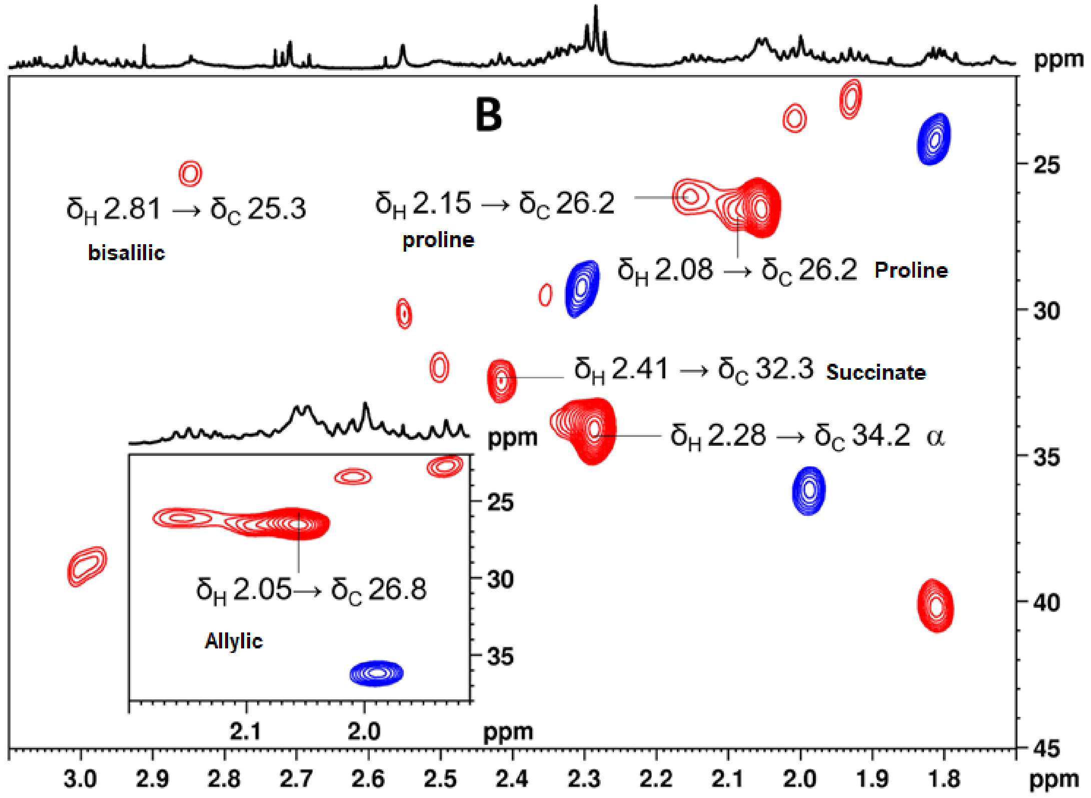

Figure S3. Ampliation from ^{1}H-^{13}C one-bond correlation map from multiplicity edited HSQC NMR experiment acquired from *Conticribra weissflogii* (^{1}H and ^{13}C, 600 and 150 MHz, D_2O) – region *B.*

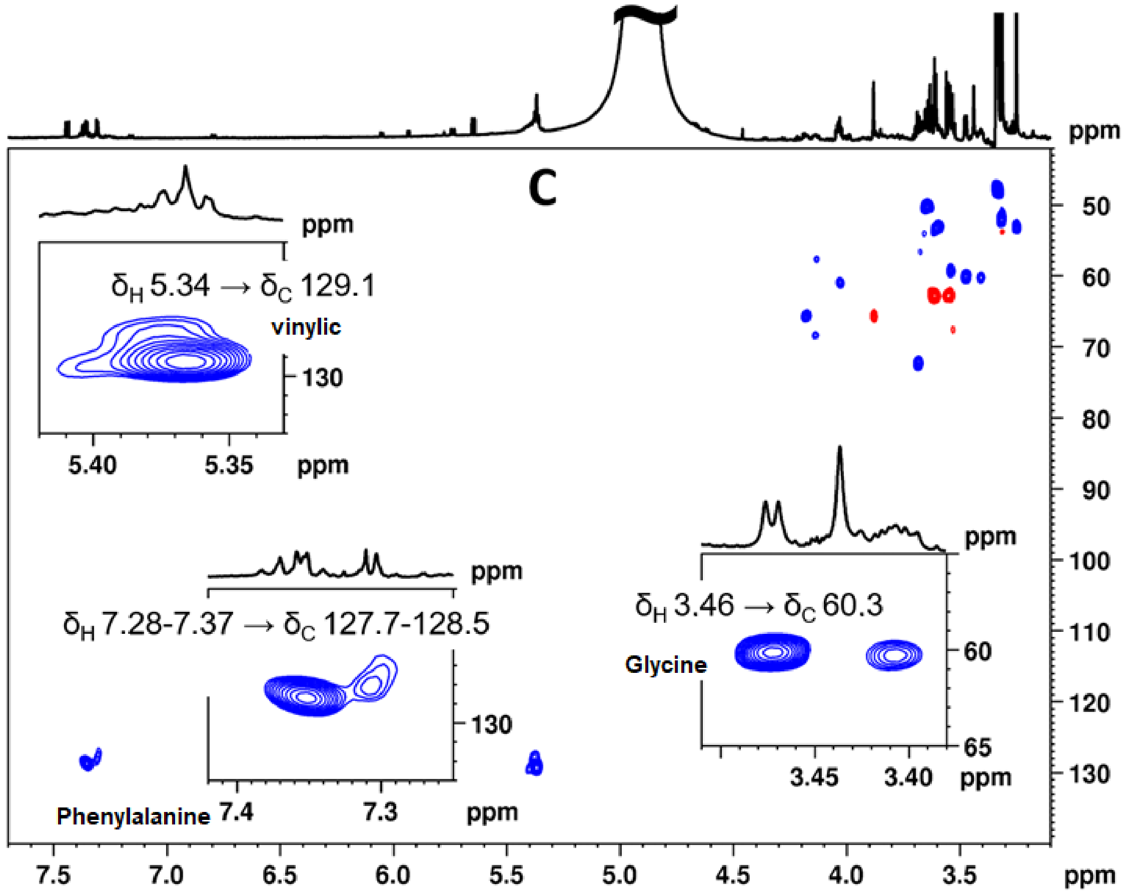

Figure S4. Ampliation from ^{1}H-^{13}C one-bond correlation map from multiplicity edited HSQC NMR experiment acquired from *Conticribra weissflogii* (^{1}H and ^{13}C, 600 and 150 MHz, D_2O) – region *C.*

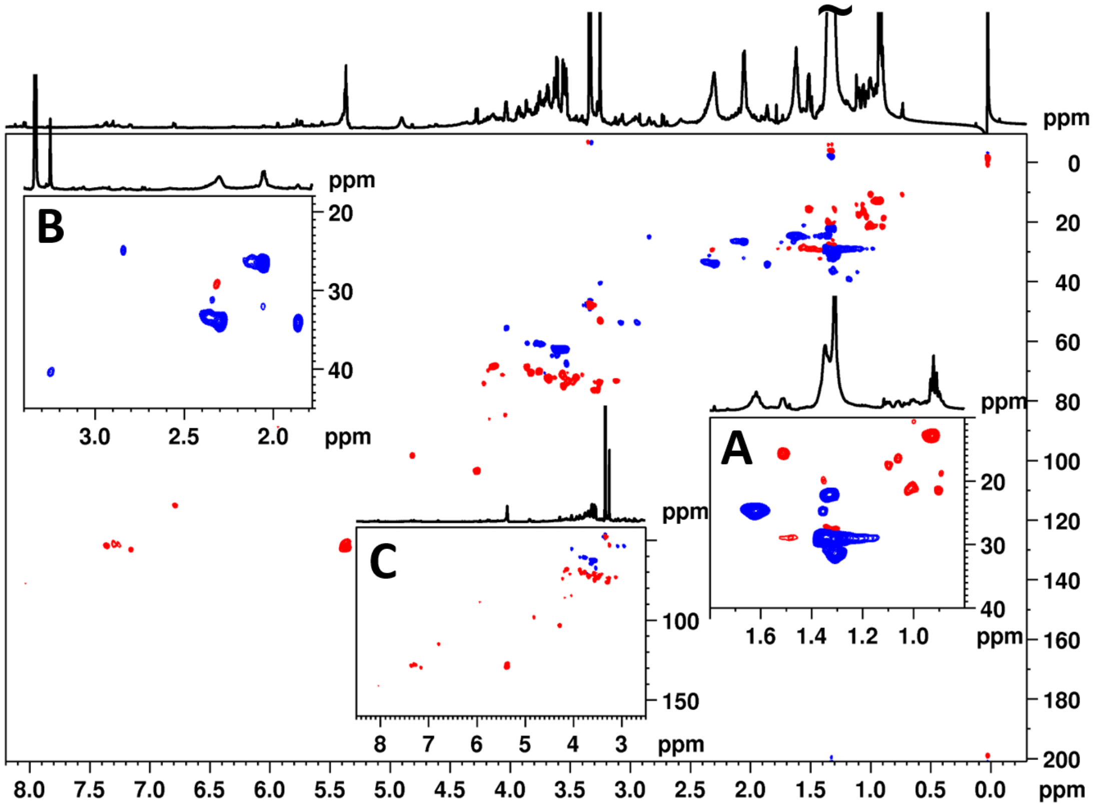

Figure S5. ^{1}H-^{13}C one-bond correlation map from multiplicity edited HSQC NMR experiment acquired from *Chaetoceros muelleri* (^{1}H and ^{13}C, 600.13 and 150.92 MHz, CD_3OD) – regions A, B, and C.

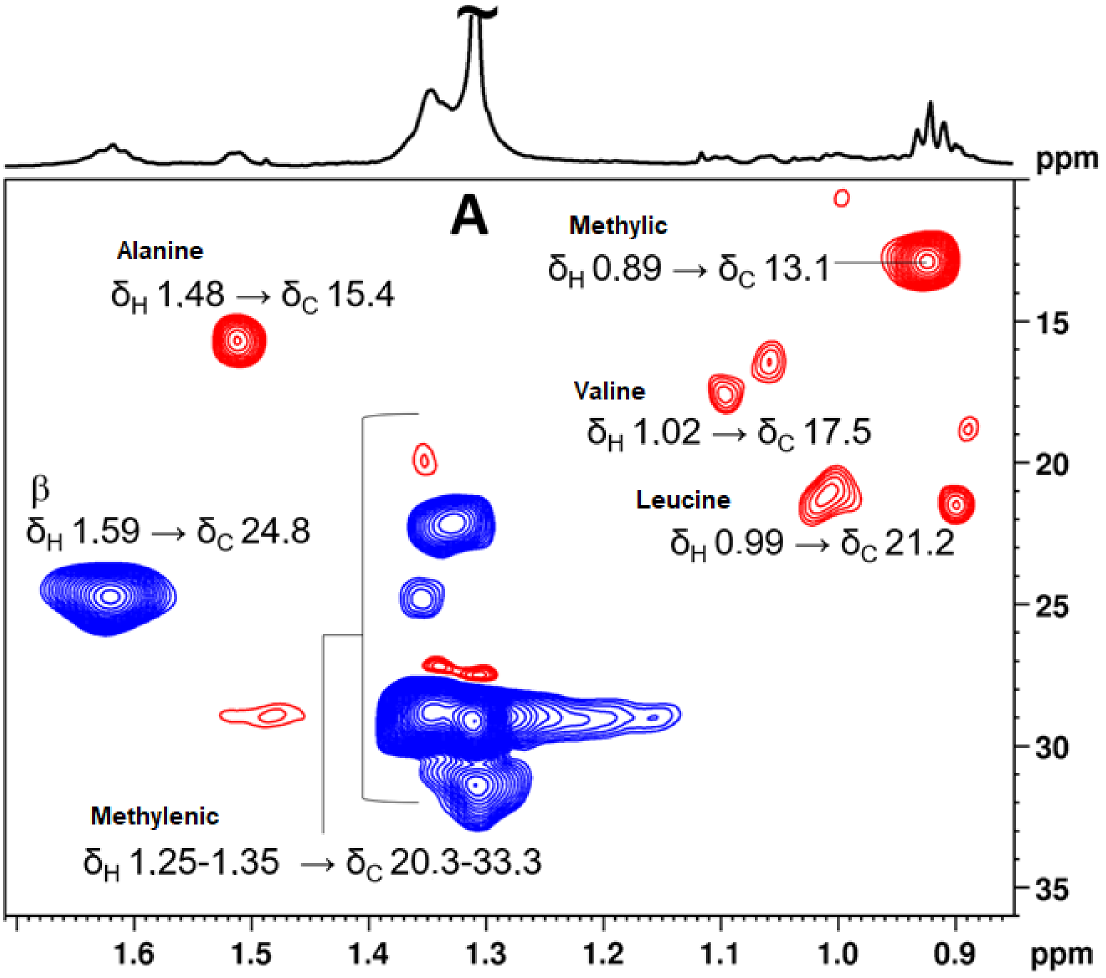

Figure S6. Ampliation from ^{1}H-^{13}C one-bond correlation map from multiplicity edited HSQC NMR experiment acquired from *Chaetoceros muelleri* (^{1}H and ^{13}C, 600.13 and 150.92 MHz, CD_3OD) – region *A*.

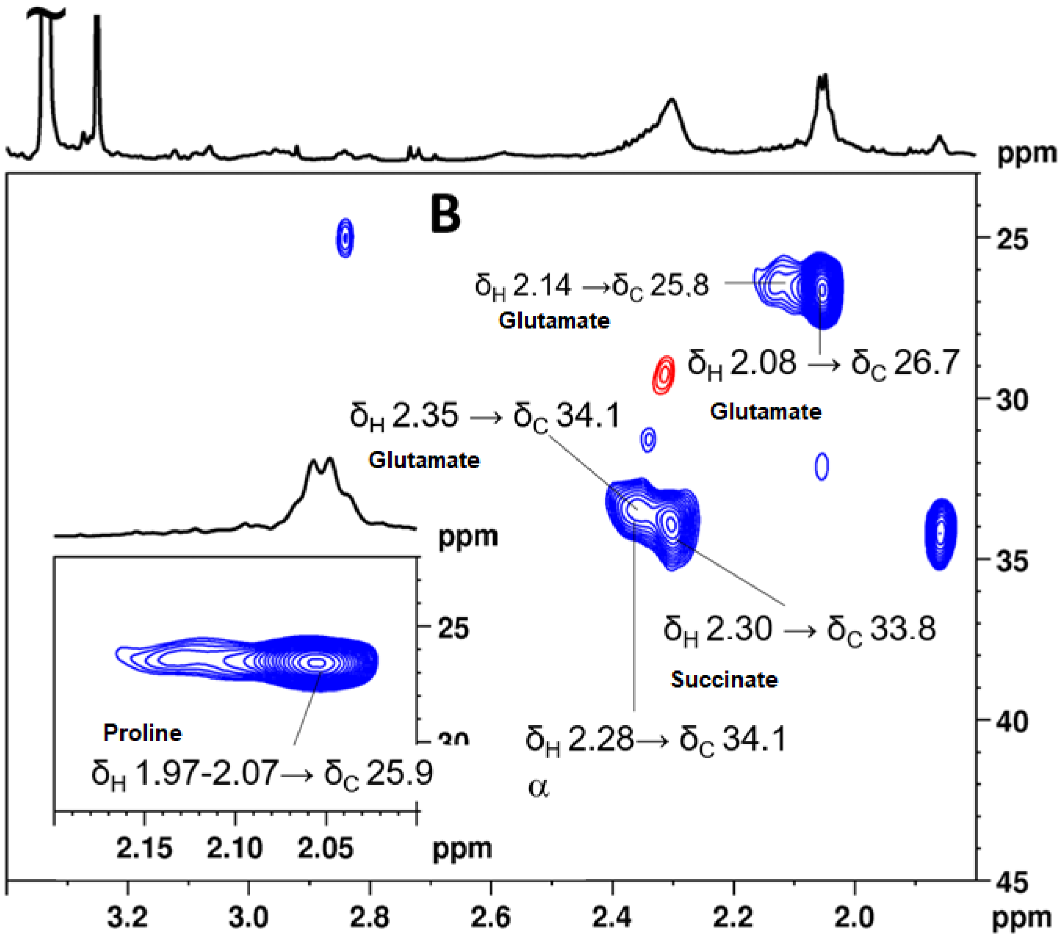

Figure S7. Ampliation from ^{1}H-^{13}C one-bond correlation map from multiplicity edited HSQC NMR experiment acquired from *Chaetoceros muelleri* (^{1}H and ^{13}C, 600.13 and 150.92 MHz, CD_3OD) – region *B*.

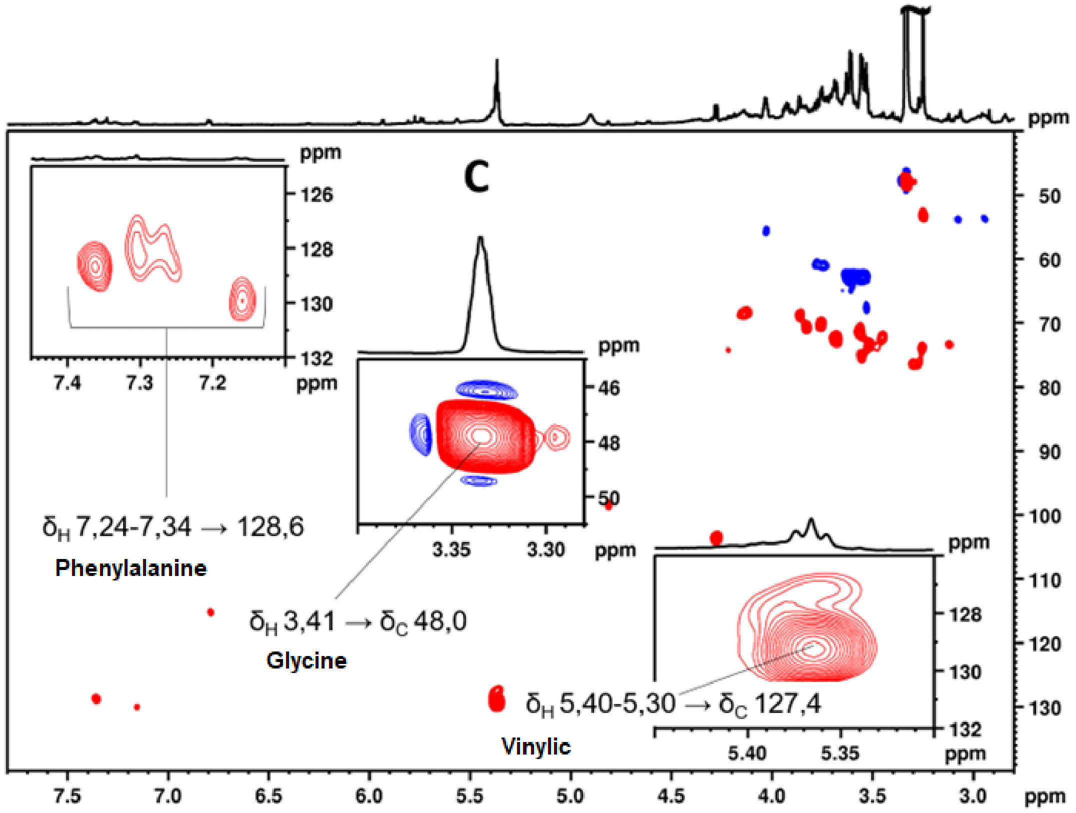

Figure S8. Ampliation from ^{1}H-^{13}C one-bond correlation map from multiplicity edited HSQC NMR experiment acquired from *Chaetoceros muelleri* (^{1}H and ^{13}C, 600.13 and 150.92 MHz, CD_3OD) – region *C*.

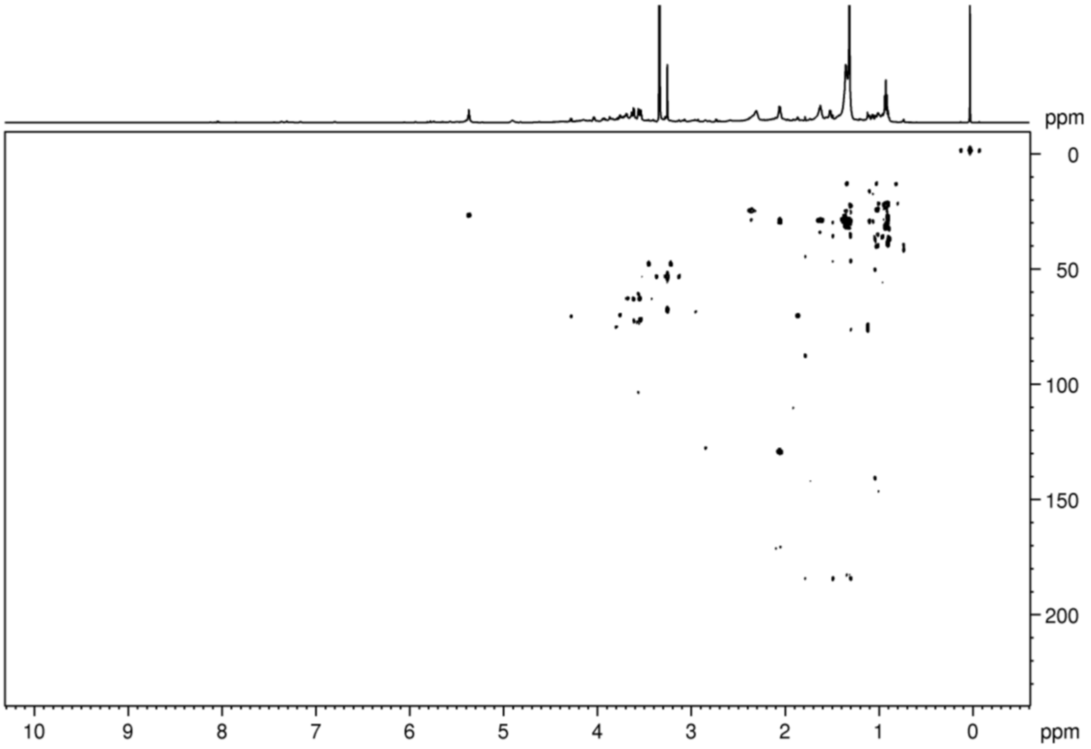

Figure S9. ^{1}H-^{13}C long-distance correlation map HMBC NMR experiment acquired from *Chaetoceros muelleri* (^{1}H and ^{13}C, 600.13 and 150.92 MHz, CD_3OD).

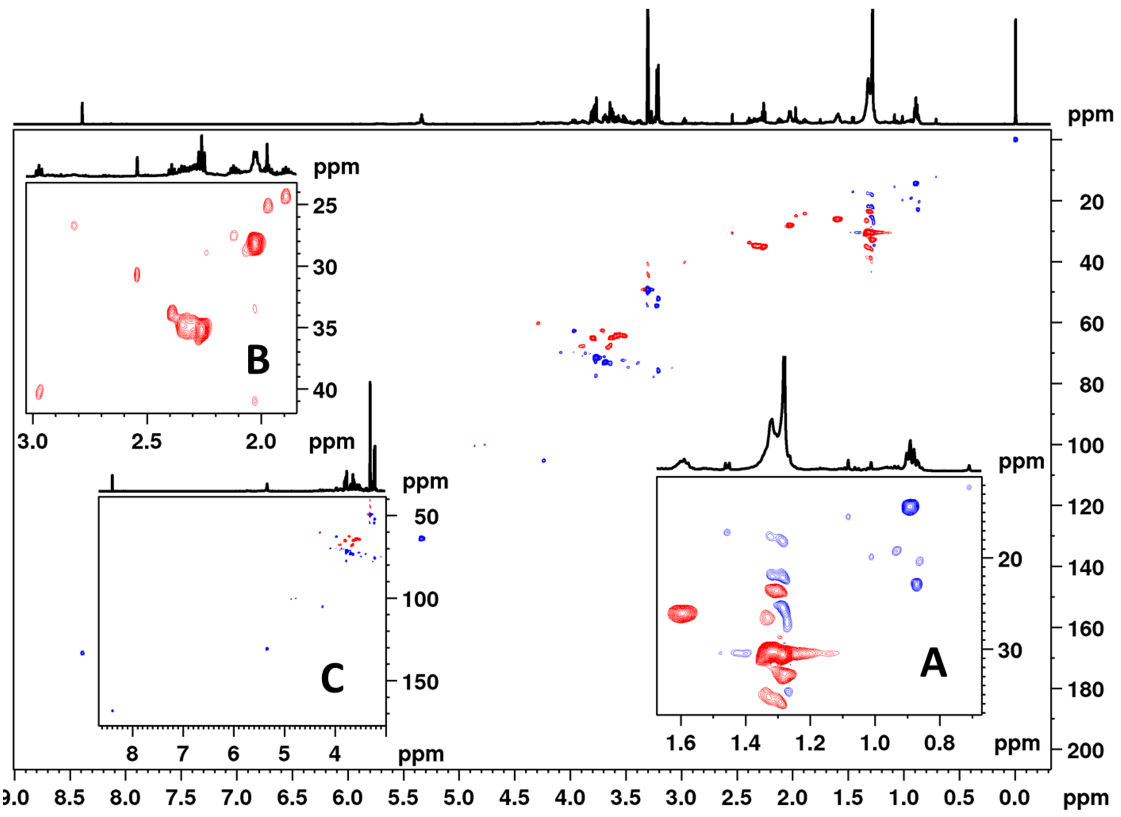

Figure S10. ^{1}H-^{13}C one-bond correlation map from multiplicity edited HSQC NMR experiment acquired from *Nannochloropsis oceanica* (^{1}H and ^{13}C, 600.13 and 150.92 MHz, CD_3OD) – regions A, B, and C.

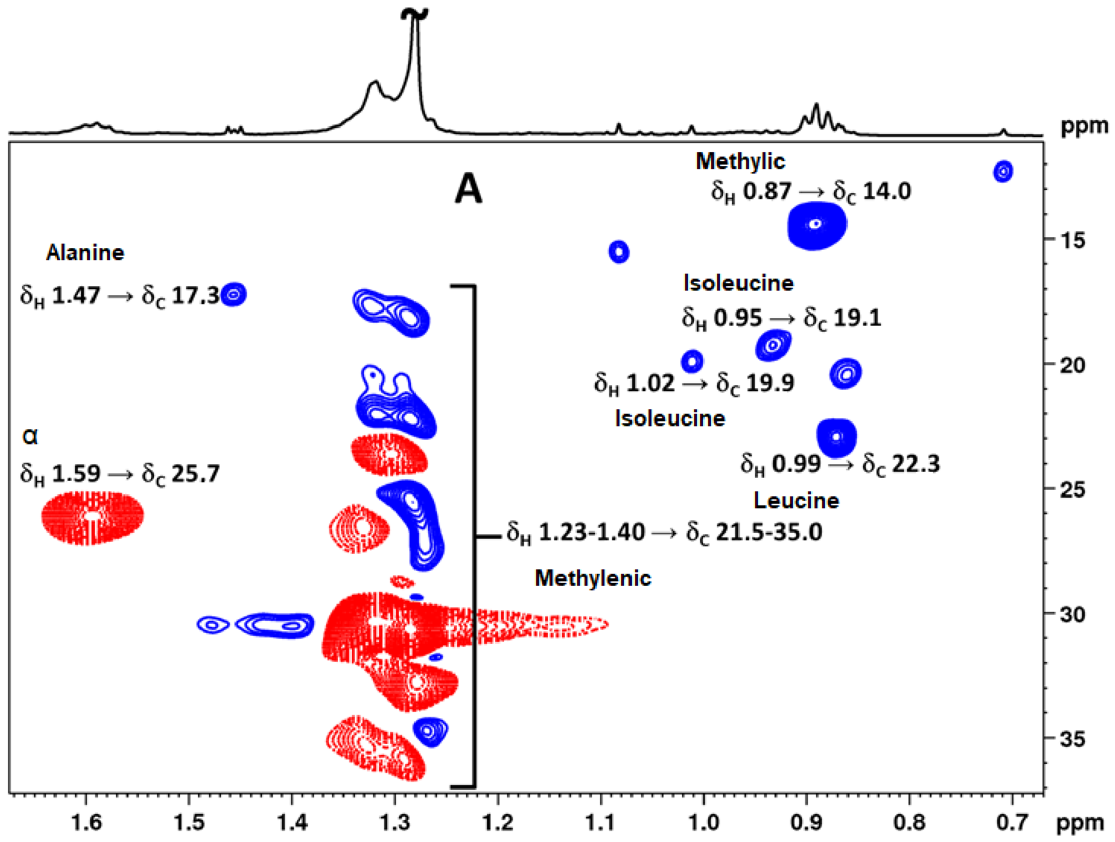

Figure S11.Ampliation from ^{1}H-^{13}C one-bond correlation map from multiplicity edited HSQC NMR experiment acquired from *Nannochloropsis oceanica* (^{1}H and ^{13}C, 600.13 and 150.92 MHz, CD_3OD) – region *A*.

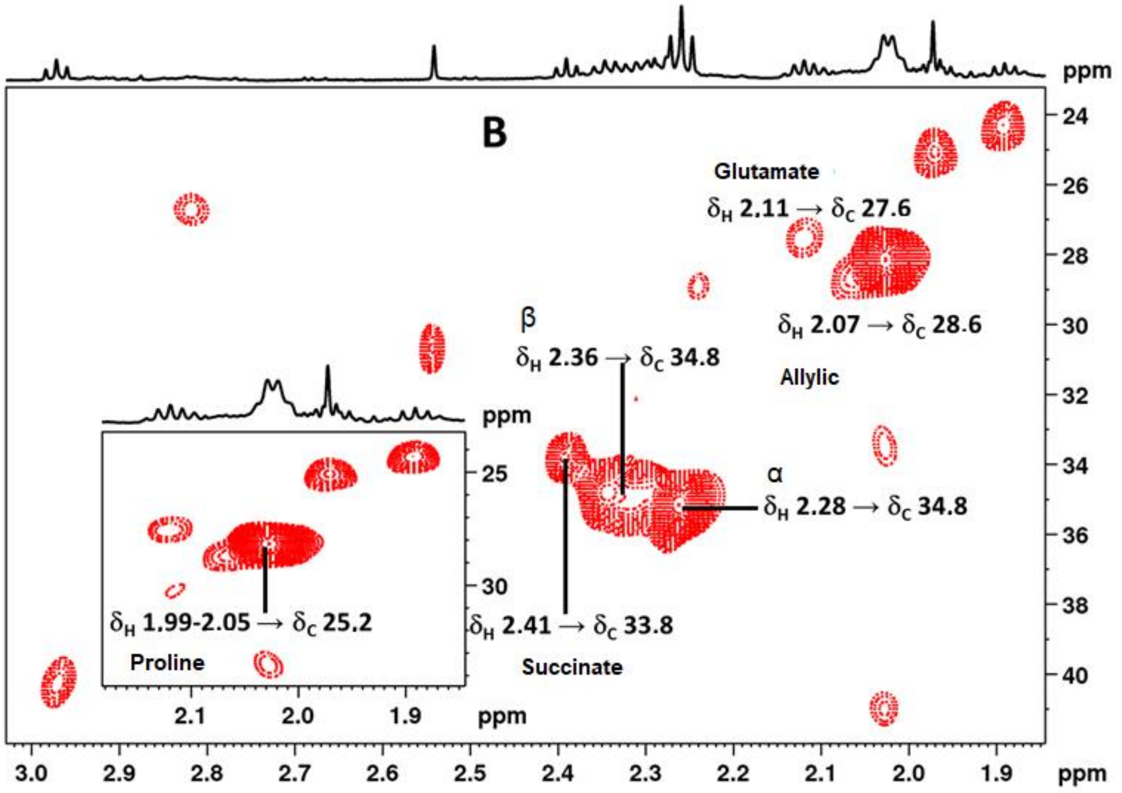

Figure S12. Ampliation from ^{1}H-^{13}C one-bond correlation map from multiplicity edited HSQC NMR experiment acquired from *Nannochloropsis oceanica* (^{1}H and ^{13}C, 600.13 and 150.92 MHz, CD_3OD) – region *B*.

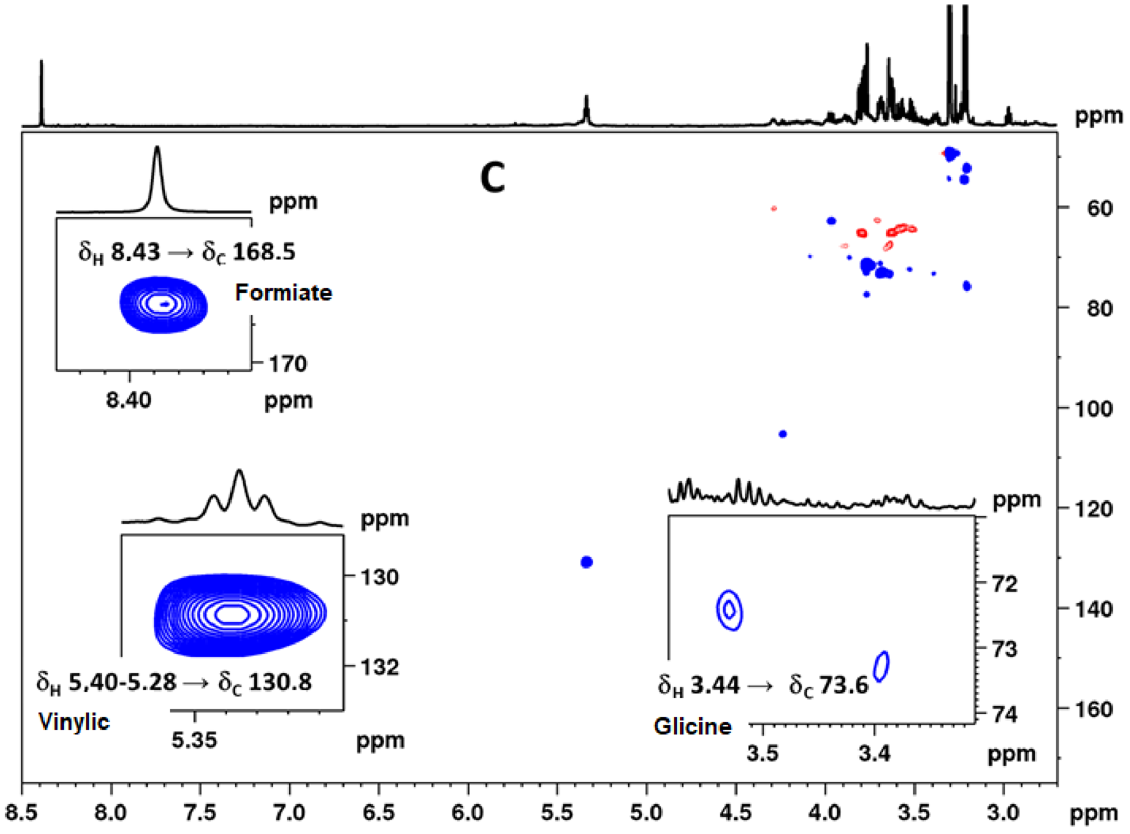

Figure S13.Ampliation from ^{1}H-^{13}C one-bond correlation map from multiplicity edited HSQC NMR experiment acquired from *Nannochloropsis oceanica* (^{1}H and ^{13}C, 600.13 and 150.92 MHz, CD_3OD) – region *C*.
